# Supplementary material for: Role of duplicate genes in determining the tissue-selectivity of hereditary diseases
Source: PLoS Genet. 2018 May 3;14(5):e1007327. doi: 10.1371/journal.pgen.1007327 (PMC5953478; doi:10.1371/journal.pgen.1007327)
Supplement: S1 Table — (DOCX) [file pgen.1007327.s001.docx]

| Disease tissue | Causal gene | Diseases Omim id | Disease Omim name |
| --- | --- | --- | --- |
| Brain | APP | 605714 | Cerebral amyloid angiopathy, Dutch, Italian, Iowa, Flemish, Arctic variants |
| Brain | ATCAY | 601238 | Ataxia, cerebellar, Cayman type |
| Brain | AUH | 250950 | 3-methylglutaconic aciduria, type I |
| Brain | CACNA1A | 108500 | Episodic ataxia, type 2 |
| Brain | CACNA1A | 183086 | Spinocerebellar ataxia 6 |
| Brain | CACNA1A | 141500 | Migraine, familial hemiplegic, 1 |
| Brain | CHMP1A | 614961 | Pontocerebellar hypoplasia, type 8 |
| Brain | CST3 | 105150 | Cerebral amyloid angiopathy |
| Brain | CTSD | 610127 | Ceroid lipofuscinosis, neuronal, 10 |
| Brain | DNAJC19 | 610198 | 3-methylglutaconic aciduria, type V |
| Brain | DNAJC5 | 162350 | Ceroid lipofuscinosis, neuronal, 4, Parry type |
| Brain | DOCK6 | 614219 | Adams-Oliver syndrome 2 |
| Brain | FGFR2 | 101200 | Apert syndrome |
| Brain | FLNA | 300049 | Heterotopia, periventricular |
| Brain | GPSM2 | 604213 | Chudley-McCullough syndrome |
| Brain | ITM2B | 176500 | Dementia, familial British |
| Brain | KIF21A | 135700 | Fibrosis of extraocular muscles, congenital, 1 |
| Brain | KIF7 | 200990 | Acrocallosal syndrome |
| Brain | MYH3 | 193700 | Arthrogryposis, distal, type 2A |
| Brain | MYO7A | 276900 | Usher syndrome, type 1B |
| Brain | OPHN1 | 300486 | Mental retardation, X-linked, with cerebellar hypoplasia and distinctive facial appearance |
| Brain | PMM2 | 212065 | Congenital disorder of glycosylation, type Ia |
| Brain | PTCH2 | 155255 | Medulloblastoma |
| Brain | SCN8A | 614306 | Cognitive impairment with or without cerebellar ataxia |
| Brain | SLC6A8 | 300352 | Cerebral creatine deficiency syndrome 1 |
| Brain | TOR1A | 128100 | Dystonia-1, torsion |
| Brain | VLDLR | 224050 | Cerebellar hypoplasia and mental retardation with or without quadrupedal locomotion 1 |
| Brain | VRK1 | 607596 | Pontocerebellar hypoplasia type 1A |
| Heart | ANK2 | 600919 | Cardiac arrhythmia, ankyrin-B-related |
| Heart | BRAF | 115150 | Cardiofaciocutaneous syndrome |
| Heart | CACNA1C | 601005 | Timothy syndrome |
| Heart | CAV3 | 192600 | Cardiomyopathy, hypertrophic, 1 |
| Heart | CSRP3 | 607482 | Cardiomyopathy, dilated, 1M |
| Heart | DES | 604765 | Cardiomyopathy, dilated, 1I |
| Heart | DMD | 302045 | Cardiomyopathy, dilated, 3B |
| Heart | ENPP1 | 208000 | Arterial calcification, generalized, of infancy, 1 |
| Heart | FBN1 | 154700 | Marfan syndrome |
| Heart | FGFR2 | 101200 | Apert syndrome |
| Heart | FLNA | 311300 | Otopalatodigital syndrome, type I |
| Heart | FLNA | 305620 | Frontometaphyseal dysplasia |
| Heart | GATA4 | 607941 | Atrial septal defect 2 |
| Heart | GATA4 | 187500 | Tetralogy of Fallot |
| Heart | GJA1 | 241550 | Hypoplastic left heart syndrome 1 |
| Heart | GJA1 | 600309 | Atrioventricular septal defect 3 |
| Heart | GJA1 | 218400 | Craniometaphyseal dysplasia, autosomal recessive |
| Heart | GNAI2 | 192605 | Ventricular tachycardia, idiopathic |
| Heart | JAG1 | 187500 | Tetralogy of Fallot |
| Heart | JUP | 601214 | Naxos disease |
| Heart | KCNJ2 | 170390 | Andersen syndrome |
| Heart | LMNA | 115200 | Cardiomyopathy, dilated, 1A |
| Heart | LMNA | 181350 | Emery-Dreifuss muscular dystrophy 2, AD |
| Heart | PRKAG2 | 600858 | Cardiomyopathy, hypertrophic 6 |
| Heart | PRKAR1A | 255960 | Myxoma, intracardiac |
| Heart | PTPN11 | 163950 | Noonan syndrome 1 |
| Heart | PTPN11 | 151100 | LEOPARD syndrome 1 |
| Heart | RYR2 | 600996 | Arrhythmogenic right ventricular dysplasia 2 |
| Heart | RYR2 | 604772 | Ventricular tachycardia, catecholaminergic polymorphic, 1 |
| Heart | SGCD | 606685 | Cardiomyopathy, dilated, 1L |
| Heart | TGFBR1 | 609192 | Loeys-Dietz syndrome 1 |
| Heart | TNNI3 | 115210 | Cardiomyopathy, familial restrictive, 1 |
| Heart | TPM1 | 115196 | Cardiomyopathy, hypertrophic, 3 |
| Heart | ZIC3 | 306955 | Congenital heart defects, nonsyndromic, 1, X-linked |
| Liver | ABHD5 | 275630 | Chanarin-Dorfman syndrome |
| Liver | CPS1 | 237300 | Carbamoylphosphate synthetase I deficiency |
| Liver | JAG1 | 118450 | Alagille syndrome |
| Liver | TF | 209300 | Atransferrinemia |
| Skeletal Muscle | ACTA1 | 161800 | Myopathy, actin, congenital, with cores |
| Skeletal Muscle | CACNA1S | 170400 | Hypokalemic periodic paralysis, type 1 |
| Skeletal Muscle | CAV3 | 606072 | Rippling muscle disease |
| Skeletal Muscle | CAV3 | 607801 | Muscular dystrophy, limb-girdle, type IC |
| Skeletal Muscle | CHRNB1 | 601462 | Myasthenic syndrome, congenital, 1A, slow-channel |
| Skeletal Muscle | CRYAB | 608810 | Myopathy, myofibrillar, 2 |
| Skeletal Muscle | DES | 601419 | Myopathy, myofibrillar, 1 |
| Skeletal Muscle | DMD | 300376 | Becker muscular dystrophy |
| Skeletal Muscle | DMD | 310200 | Duchenne muscular dystrophy |
| Skeletal Muscle | DYSF | 253601 | Muscular dystrophy, limb-girdle, type 2B |
| Skeletal Muscle | DYSF | 254130 | Miyoshi muscular dystrophy 1 |
| Skeletal Muscle | DYSF | 606768 | Myopathy, distal, with anterior tibial onset |
| Skeletal Muscle | EGR2 | 607678 | Charcot-Marie-Tooth disease, type 1D |
| Skeletal Muscle | KIF21A | 135700 | Fibrosis of extraocular muscles, congenital, 1 |
| Skeletal Muscle | LAMA2 | 607855 | Muscular dystrophy, congenital merosin-deficient |
| Skeletal Muscle | LMNA | 159001 | Muscular dystrophy, limb-girdle, type 1B |
| Skeletal Muscle | LMNA | 181350 | Emery-Dreifuss muscular dystrophy 2, AD |
| Skeletal Muscle | MYH3 | 601680 | Arthrogryposis, distal, type 2B |
| Skeletal Muscle | RYR1 | 145600 | King-Denborough syndrome |
| Skeletal Muscle | SGCD | 601287 | Muscular dystrophy, limb-girdle, type 2F |
| Skeletal Muscle | SLC25A4 | 609283 | Progressive external ophthalmoplegia with mitochondrial DNA deletions, autosomal dominant 2 |
| Skeletal Muscle | TPM2 | 601680 | Arthrogryposis, distal, type 2B |
| Skin | ATP2A2 | 124200 | Darier disease |
| Skin | COL1A1 | 130000 | Ehlers-Danlos syndrome, classic |
| Skin | FGFR2 | 123790 | Beare-Stevenson cutis gyrata syndrome |
| Skin | KRT1 | 113800 | Epidermolytic hyperkeratosis |
| Skin | KRT1 | 607602 | Ichthyosis, cyclic, with epidermolytic hyperkeratosis |
| Skin | KRT1 | 144200 | Palmoplantar keratoderma, epidermolytic |
| Skin | KRT1 | 607654 | Keratosis palmoplantaris striata III |
| Skin | KRT1 | 146590 | Ichthyosis histrix, Curth-Macklin type |
| Skin | KRT10 | 600962 | Palmoplantar keratoderma, nonepidermolytic |
| Skin | KRT10 | 113800 | Epidermolytic hyperkeratosis |
| Skin | KRT14 | 131800 | Epidermolysis bullosa of hands and feet |
| Skin | KRT14 | 601001 | Epidermolysis bullosa simplex, recessive 1 |
| Skin | KRT14 | 131760 | Epidermolysis bullosa simplex, Dowling-Meara type |
| Skin | KRT14 | 131900 | Epidermolysis bullosa simplex, Koebner type |
| Skin | KRT17 | 184500 | Steatocystoma multiplex |
| Skin | KRT5 | 131800 | Epidermolysis bullosa of hands and feet |
| Skin | KRT5 | 601001 | Epidermolysis bullosa simplex, recessive 1 |
| Skin | KRT5 | 131760 | Epidermolysis bullosa simplex, Dowling-Meara type |
| Skin | KRT5 | 131900 | Epidermolysis bullosa simplex, Koebner type |
| Skin | KRT5 | 131960 | Epidermolysis bullosa simplex-MP |
| Skin | LAMA3 | 226650 | Epidermolysis bullosa, generalized atrophic benign |
| Skin | LAMA3 | 226700 | Epidermolysis bullosa, junctional, Herlitz type |
| Skin | LAMC2 | 226650 | Epidermolysis bullosa, junctional, non-Herlitz type |
| Skin | LAMC2 | 226700 | Epidermolysis bullosa, junctional, Herlitz type |
| Skin | LMNA | 176670 | Hutchinson-Gilford progeria |
| Skin | MITF | 103470 | Waardenburg syndrome/ocular albinism, digenic |
| Skin | MITF | 193510 | Waardenburg syndrome, type 2A |
| Skin | NAGA | 609242 | Kanzaki disease |
| Skin | NOTCH3 | 615293 | Myofibromatosis, infantile 2 |
| Skin | PTCH1 | 109400 | Basal cell nevus syndrome |
| Skin | PTCH2 | 109400 | Basal cell nevus syndrome |
| Skin | SNAI2 | 608890 | Waardenburg syndrome, type 2D |
| Skin | SNAI2 | 172800 | Piebaldism |
| Skin | TYRP1 | 203290 | Albinism, oculocutaneous, type III |
| Testis | SOX9 | 114290 | Acampomelic campomelic dysplasia |
| Thyroid | NTRK1 | 155240 | Medullary thyroid carcinoma, familial |
