# Supplementary material for: Role of duplicate genes in determining the tissue-selectivity of hereditary diseases
Source: PLoS Genet. 2018 May 3;14(5):e1007327. doi: 10.1371/journal.pgen.1007327 (PMC5953478; doi:10.1371/journal.pgen.1007327)
Supplement: S2 Table — (DOCX) [file pgen.1007327.s002.docx]

| **Disease tissue** | **Disease gene** | **Paralog** |
| --- | --- | --- |
| Brain | ENSG00000066468 | ENSG00000068078 |
| Brain | ENSG00000066468 | ENSG00000077782 |
| Brain | ENSG00000066468 | ENSG00000160867 |
| Brain | ENSG00000079482 | ENSG00000071205 |
| Brain | ENSG00000079482 | ENSG00000145819 |
| Brain | ENSG00000079482 | ENSG00000165895 |
| Brain | ENSG00000100749 | ENSG00000028116 |
| Brain | ENSG00000101152 | ENSG00000163793 |
| Brain | ENSG00000101439 | ENSG00000170369 |
| Brain | ENSG00000109063 | ENSG00000078814 |
| Brain | ENSG00000109063 | ENSG00000197616 |
| Brain | ENSG00000109063 | ENSG00000144821 |
| Brain | ENSG00000109063 | ENSG00000125414 |
| Brain | ENSG00000109063 | ENSG00000109061 |
| Brain | ENSG00000117425 | ENSG00000185920 |
| Brain | ENSG00000117984 | ENSG00000131400 |
| Brain | ENSG00000121957 | ENSG00000160360 |
| Brain | ENSG00000130158 | ENSG00000116641 |
| Brain | ENSG00000130158 | ENSG00000107099 |
| Brain | ENSG00000130821 | ENSG00000131389 |
| Brain | ENSG00000130821 | ENSG00000157103 |
| Brain | ENSG00000130821 | ENSG00000010379 |
| Brain | ENSG00000130821 | ENSG00000111181 |
| Brain | ENSG00000130821 | ENSG00000132164 |
| Brain | ENSG00000131165 | ENSG00000255112 |
| Brain | ENSG00000136156 | ENSG00000135916 |
| Brain | ENSG00000136827 | ENSG00000136816 |
| Brain | ENSG00000137474 | ENSG00000169994 |
| Brain | ENSG00000139116 | ENSG00000116852 |
| Brain | ENSG00000140650 | ENSG00000100417 |
| Brain | ENSG00000141837 | ENSG00000148408 |
| Brain | ENSG00000141837 | ENSG00000198216 |
| Brain | ENSG00000142192 | ENSG00000084234 |
| Brain | ENSG00000147852 | ENSG00000130164 |
| Brain | ENSG00000147852 | ENSG00000157193 |
| Brain | ENSG00000148090 | ENSG00000121310 |
| Brain | ENSG00000166813 | ENSG00000165115 |
| Brain | ENSG00000167654 | ENSG00000140299 |
| Brain | ENSG00000196569 | ENSG00000101680 |
| Brain | ENSG00000196876 | ENSG00000169432 |
| Brain | ENSG00000196876 | ENSG00000153253 |
| Brain | ENSG00000196876 | ENSG00000136531 |
| Brain | ENSG00000196876 | ENSG00000136546 |
| Brain | ENSG00000196876 | ENSG00000144285 |
| Brain | ENSG00000196876 | ENSG00000183873 |
| Brain | ENSG00000196876 | ENSG00000168356 |
| Brain | ENSG00000196876 | ENSG00000007314 |
| Brain | ENSG00000196924 | ENSG00000136068 |
| Brain | ENSG00000196924 | ENSG00000128591 |
| Brain | ENSG00000205981 | ENSG00000120675 |
| Heart | ENSG00000066468 | ENSG00000160867 |
| Heart | ENSG00000101384 | ENSG00000184916 |
| Heart | ENSG00000106617 | ENSG00000181929 |
| Heart | ENSG00000106799 | ENSG00000135503 |
| Heart | ENSG00000106799 | ENSG00000115170 |
| Heart | ENSG00000106799 | ENSG00000107779 |
| Heart | ENSG00000106799 | ENSG00000139567 |
| Heart | ENSG00000106799 | ENSG00000138696 |
| Heart | ENSG00000106799 | ENSG00000123612 |
| Heart | ENSG00000108946 | ENSG00000188191 |
| Heart | ENSG00000114353 | ENSG00000156052 |
| Heart | ENSG00000114353 | ENSG00000087258 |
| Heart | ENSG00000114353 | ENSG00000065135 |
| Heart | ENSG00000114353 | ENSG00000088256 |
| Heart | ENSG00000114353 | ENSG00000128266 |
| Heart | ENSG00000114353 | ENSG00000127955 |
| Heart | ENSG00000114353 | ENSG00000060558 |
| Heart | ENSG00000114353 | ENSG00000156049 |
| Heart | ENSG00000123700 | ENSG00000121361 |
| Heart | ENSG00000123700 | ENSG00000184185 |
| Heart | ENSG00000123700 | ENSG00000182324 |
| Heart | ENSG00000123700 | ENSG00000187486 |
| Heart | ENSG00000123700 | ENSG00000162989 |
| Heart | ENSG00000123700 | ENSG00000120457 |
| Heart | ENSG00000123700 | ENSG00000153822 |
| Heart | ENSG00000123700 | ENSG00000168135 |
| Heart | ENSG00000123700 | ENSG00000162728 |
| Heart | ENSG00000123700 | ENSG00000157542 |
| Heart | ENSG00000129170 | ENSG00000159176 |
| Heart | ENSG00000129991 | ENSG00000130598 |
| Heart | ENSG00000129991 | ENSG00000159173 |
| Heart | ENSG00000136574 | ENSG00000141448 |
| Heart | ENSG00000136574 | ENSG00000130700 |
| Heart | ENSG00000140416 | ENSG00000143549 |
| Heart | ENSG00000140416 | ENSG00000167460 |
| Heart | ENSG00000140416 | ENSG00000198467 |
| Heart | ENSG00000145362 | ENSG00000151150 |
| Heart | ENSG00000151067 | ENSG00000157388 |
| Heart | ENSG00000151067 | ENSG00000102001 |
| Heart | ENSG00000151067 | ENSG00000081248 |
| Heart | ENSG00000152661 | ENSG00000187513 |
| Heart | ENSG00000152661 | ENSG00000121743 |
| Heart | ENSG00000156925 | ENSG00000043355 |
| Heart | ENSG00000156925 | ENSG00000139800 |
| Heart | ENSG00000156925 | ENSG00000152977 |
| Heart | ENSG00000156925 | ENSG00000174963 |
| Heart | ENSG00000157764 | ENSG00000078061 |
| Heart | ENSG00000157764 | ENSG00000132155 |
| Heart | ENSG00000160789 | ENSG00000176619 |
| Heart | ENSG00000160789 | ENSG00000113368 |
| Heart | ENSG00000166147 | ENSG00000142449 |
| Heart | ENSG00000166147 | ENSG00000138829 |
| Heart | ENSG00000170624 | ENSG00000102683 |
| Heart | ENSG00000170624 | ENSG00000185053 |
| Heart | ENSG00000173801 | ENSG00000168036 |
| Heart | ENSG00000175084 | ENSG00000026025 |
| Heart | ENSG00000175084 | ENSG00000131095 |
| Heart | ENSG00000175084 | ENSG00000135406 |
| Heart | ENSG00000175084 | ENSG00000148798 |
| Heart | ENSG00000179295 | ENSG00000111679 |
| Heart | ENSG00000182533 | ENSG00000105974 |
| Heart | ENSG00000196924 | ENSG00000136068 |
| Heart | ENSG00000196924 | ENSG00000128591 |
| Heart | ENSG00000197594 | ENSG00000136960 |
| Heart | ENSG00000197594 | ENSG00000154269 |
| Heart | ENSG00000198626 | ENSG00000196218 |
| Heart | ENSG00000198626 | ENSG00000198838 |
| Heart | ENSG00000198947 | ENSG00000152818 |
| Liver | ENSG00000011198 | ENSG00000100439 |
| Liver | ENSG00000021826 | ENSG00000084774 |
| Liver | ENSG00000091513 | ENSG00000163975 |
| Liver | ENSG00000101384 | ENSG00000184916 |
| Muscle | ENSG00000081248 | ENSG00000151067 |
| Muscle | ENSG00000081248 | ENSG00000141837 |
| Muscle | ENSG00000109063 | ENSG00000092054 |
| Muscle | ENSG00000109063 | ENSG00000133392 |
| Muscle | ENSG00000109063 | ENSG00000100345 |
| Muscle | ENSG00000109063 | ENSG00000133026 |
| Muscle | ENSG00000109063 | ENSG00000078814 |
| Muscle | ENSG00000109063 | ENSG00000197616 |
| Muscle | ENSG00000109063 | ENSG00000144821 |
| Muscle | ENSG00000109063 | ENSG00000125414 |
| Muscle | ENSG00000109063 | ENSG00000109061 |
| Muscle | ENSG00000109846 | ENSG00000106211 |
| Muscle | ENSG00000109846 | ENSG00000004776 |
| Muscle | ENSG00000122877 | ENSG00000179388 |
| Muscle | ENSG00000135636 | ENSG00000138119 |
| Muscle | ENSG00000139116 | ENSG00000116852 |
| Muscle | ENSG00000143632 | ENSG00000184009 |
| Muscle | ENSG00000143632 | ENSG00000075624 |
| Muscle | ENSG00000143632 | ENSG00000107796 |
| Muscle | ENSG00000143632 | ENSG00000163017 |
| Muscle | ENSG00000143632 | ENSG00000184378 |
| Muscle | ENSG00000143632 | ENSG00000159251 |
| Muscle | ENSG00000151729 | ENSG00000169100 |
| Muscle | ENSG00000151729 | ENSG00000005022 |
| Muscle | ENSG00000160789 | ENSG00000176619 |
| Muscle | ENSG00000160789 | ENSG00000113368 |
| Muscle | ENSG00000170175 | ENSG00000160716 |
| Muscle | ENSG00000170624 | ENSG00000102683 |
| Muscle | ENSG00000170624 | ENSG00000185053 |
| Muscle | ENSG00000175084 | ENSG00000026025 |
| Muscle | ENSG00000175084 | ENSG00000131095 |
| Muscle | ENSG00000175084 | ENSG00000135406 |
| Muscle | ENSG00000175084 | ENSG00000148798 |
| Muscle | ENSG00000182533 | ENSG00000105974 |
| Muscle | ENSG00000196218 | ENSG00000198838 |
| Muscle | ENSG00000196218 | ENSG00000198626 |
| Muscle | ENSG00000196569 | ENSG00000101680 |
| Muscle | ENSG00000198467 | ENSG00000167460 |
| Muscle | ENSG00000198467 | ENSG00000143549 |
| Muscle | ENSG00000198467 | ENSG00000140416 |
| Muscle | ENSG00000198947 | ENSG00000152818 |
| Skin | ENSG00000019549 | ENSG00000185669 |
| Skin | ENSG00000019549 | ENSG00000124216 |
| Skin | ENSG00000053747 | ENSG00000130702 |
| Skin | ENSG00000058085 | ENSG00000135862 |
| Skin | ENSG00000066468 | ENSG00000068078 |
| Skin | ENSG00000066468 | ENSG00000077782 |
| Skin | ENSG00000066468 | ENSG00000160867 |
| Skin | ENSG00000074181 | ENSG00000134250 |
| Skin | ENSG00000074181 | ENSG00000148400 |
| Skin | ENSG00000107165 | ENSG00000080166 |
| Skin | ENSG00000108821 | ENSG00000204248 |
| Skin | ENSG00000108821 | ENSG00000080573 |
| Skin | ENSG00000108821 | ENSG00000164692 |
| Skin | ENSG00000108821 | ENSG00000168542 |
| Skin | ENSG00000108821 | ENSG00000130635 |
| Skin | ENSG00000108821 | ENSG00000204262 |
| Skin | ENSG00000108821 | ENSG00000060718 |
| Skin | ENSG00000117425 | ENSG00000185920 |
| Skin | ENSG00000128422 | ENSG00000171401 |
| Skin | ENSG00000128422 | ENSG00000186395 |
| Skin | ENSG00000128422 | ENSG00000171345 |
| Skin | ENSG00000128422 | ENSG00000186847 |
| Skin | ENSG00000128422 | ENSG00000186832 |
| Skin | ENSG00000128422 | ENSG00000171346 |
| Skin | ENSG00000128422 | ENSG00000131737 |
| Skin | ENSG00000128422 | ENSG00000094796 |
| Skin | ENSG00000128422 | ENSG00000167916 |
| Skin | ENSG00000160789 | ENSG00000176619 |
| Skin | ENSG00000160789 | ENSG00000113368 |
| Skin | ENSG00000167768 | ENSG00000186081 |
| Skin | ENSG00000167768 | ENSG00000172867 |
| Skin | ENSG00000167768 | ENSG00000170477 |
| Skin | ENSG00000167768 | ENSG00000205420 |
| Skin | ENSG00000167768 | ENSG00000170421 |
| Skin | ENSG00000167768 | ENSG00000135480 |
| Skin | ENSG00000167768 | ENSG00000185640 |
| Skin | ENSG00000167768 | ENSG00000185479 |
| Skin | ENSG00000167768 | ENSG00000170486 |
| Skin | ENSG00000167768 | ENSG00000170484 |
| Skin | ENSG00000174437 | ENSG00000074370 |
| Skin | ENSG00000174437 | ENSG00000196296 |
| Skin | ENSG00000185920 | ENSG00000117425 |
| Skin | ENSG00000186081 | ENSG00000167768 |
| Skin | ENSG00000186081 | ENSG00000172867 |
| Skin | ENSG00000186081 | ENSG00000170477 |
| Skin | ENSG00000186081 | ENSG00000205420 |
| Skin | ENSG00000186081 | ENSG00000170421 |
| Skin | ENSG00000186081 | ENSG00000135480 |
| Skin | ENSG00000186081 | ENSG00000185640 |
| Skin | ENSG00000186081 | ENSG00000185479 |
| Skin | ENSG00000186081 | ENSG00000170486 |
| Skin | ENSG00000186081 | ENSG00000170484 |
| Skin | ENSG00000186395 | ENSG00000171401 |
| Skin | ENSG00000186395 | ENSG00000186847 |
| Skin | ENSG00000186395 | ENSG00000128422 |
| Skin | ENSG00000186395 | ENSG00000186832 |
| Skin | ENSG00000186395 | ENSG00000171346 |
| Skin | ENSG00000186395 | ENSG00000167916 |
| Skin | ENSG00000186395 | ENSG00000204897 |
| Skin | ENSG00000186395 | ENSG00000171446 |
| Skin | ENSG00000186847 | ENSG00000171401 |
| Skin | ENSG00000186847 | ENSG00000171345 |
| Skin | ENSG00000186847 | ENSG00000186395 |
| Skin | ENSG00000186847 | ENSG00000128422 |
| Skin | ENSG00000186847 | ENSG00000186832 |
| Skin | ENSG00000186847 | ENSG00000094796 |
| Skin | ENSG00000186847 | ENSG00000171346 |
| Skin | ENSG00000186847 | ENSG00000131737 |
| Skin | ENSG00000186847 | ENSG00000167916 |
| Skin | ENSG00000186847 | ENSG00000204897 |
| Skin | ENSG00000186847 | ENSG00000171446 |
| Skin | ENSG00000187098 | ENSG00000068323 |
| Skin | ENSG00000198951 | ENSG00000102393 |
| Testis | ENSG00000125398 | ENSG00000005513 |
| Testis | ENSG00000125398 | ENSG00000100146 |
| Thyroid | ENSG00000198400 | ENSG00000148053 |
| Thyroid | ENSG00000198400 | ENSG00000140538 |
