# Supplementary figures and images for: Role of duplicate genes in determining the tissue-selectivity of hereditary diseases
Source: PLoS Genet. 2018 May 3;14(5):e1007327. doi: 10.1371/journal.pgen.1007327 (PMC5953478; doi:10.1371/journal.pgen.1007327)

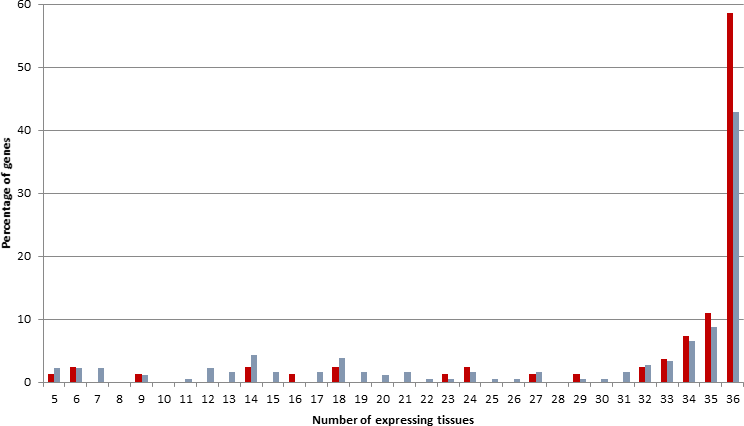

Supplement: S1 Fig — (TIF) [file pgen.1007327.s004.tif]

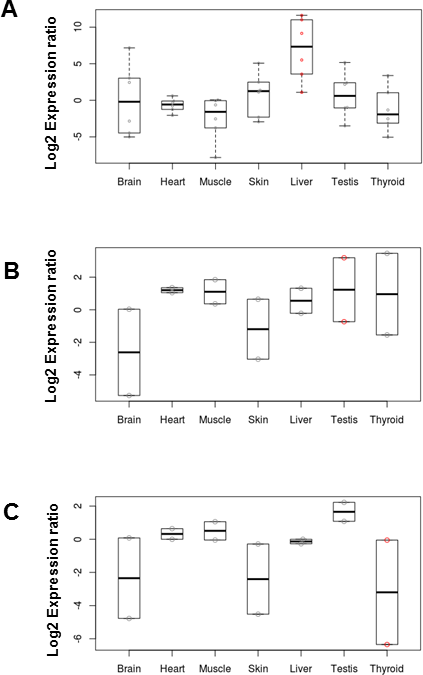

Supplement: S2 Fig — Each point represents the ratio observed in the disease tissue (red) and in an unaffected tissue (gray). The panels show genes causal for diseases manifesting in the liver (A), testis (B), thyroid (C). (TIF) [file pgen.1007327.s005.tif]

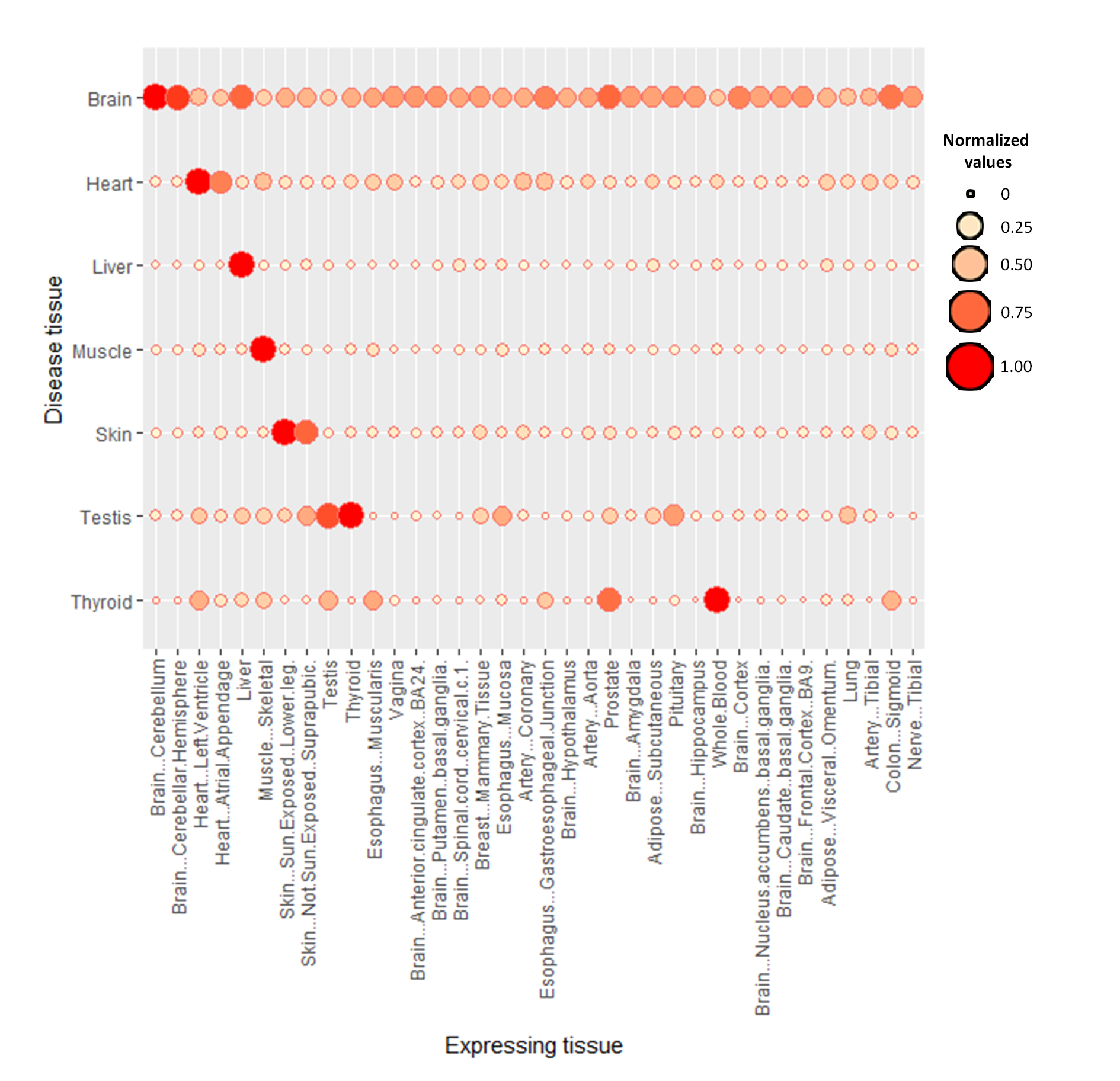

Supplement: S3 Fig — Each row corresponds to genes causal for diseases that manifest in the tissue designated on the left, and represents the median ratios per tissue normalized to the maximum in that row. In each row, the median ratios in the disease tissue are highest. All 36 tissues with 5 samples or more are shown. The imbalanced expression of genes causal for brain diseases and their paralogs is at comparatively high levels in multiple tissues mainly due to two causal genes, CST3 and CTSD, that were associated with 3 and 6 paralogs, respectively, and were expressed at relatively high levels in multiple tissues. (TIF) [file pgen.1007327.s006.tif]

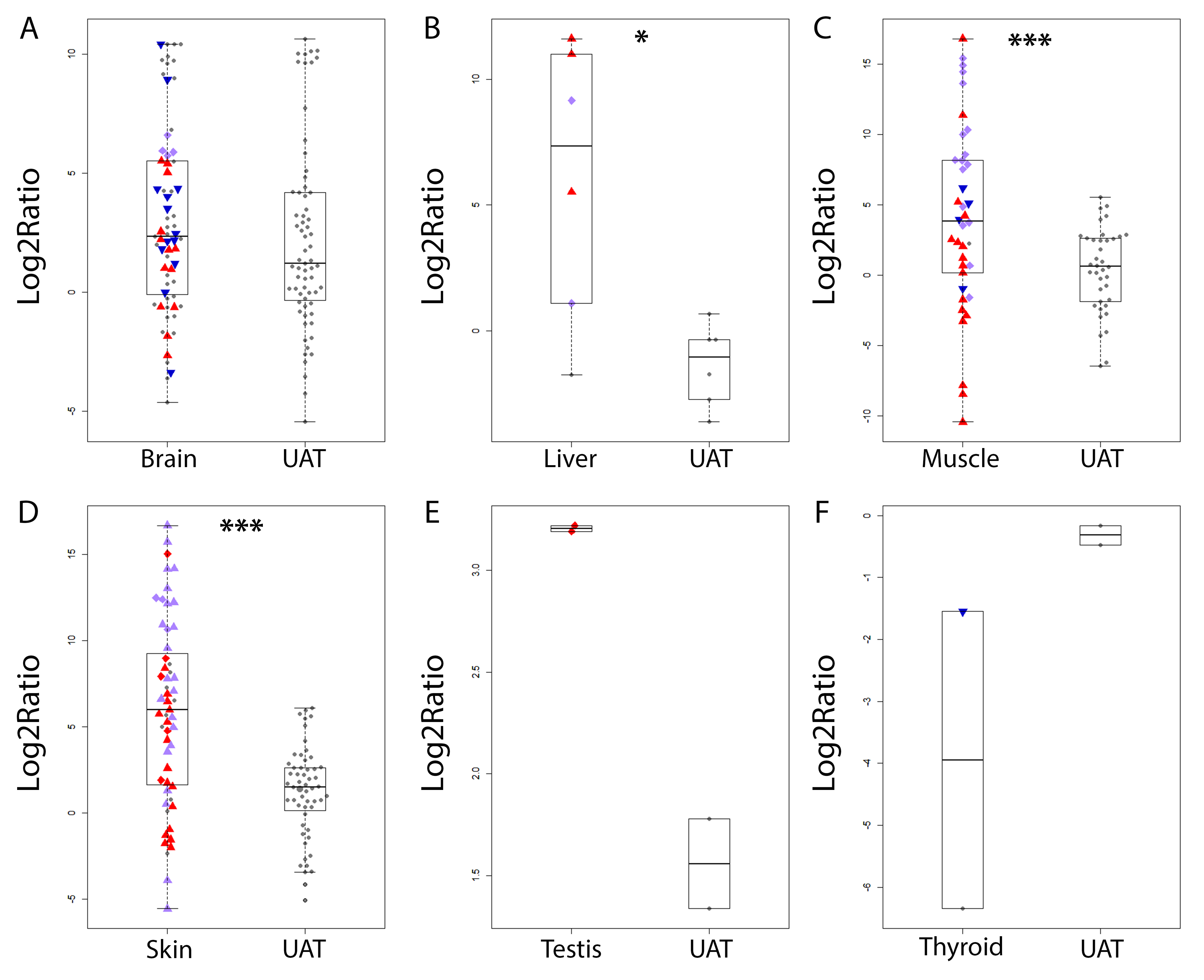

Supplement: S4 Fig — Each point represents the ratio of a specific pair in their disease tissue (DT) and unaffected tissues (UAT). Colors indicate whether in the disease tissue the causal gene was significantly over-expressed (red), the paralog was significantly under-expressed (blue), both co-occurred (purple), or none occurred (gray). The panels show genes causal for diseases manifesting in the brain (A), liver (B), muscle (C), Skin (D), testis (E), thyroid(F). (TIF) [file pgen.1007327.s007.tif]

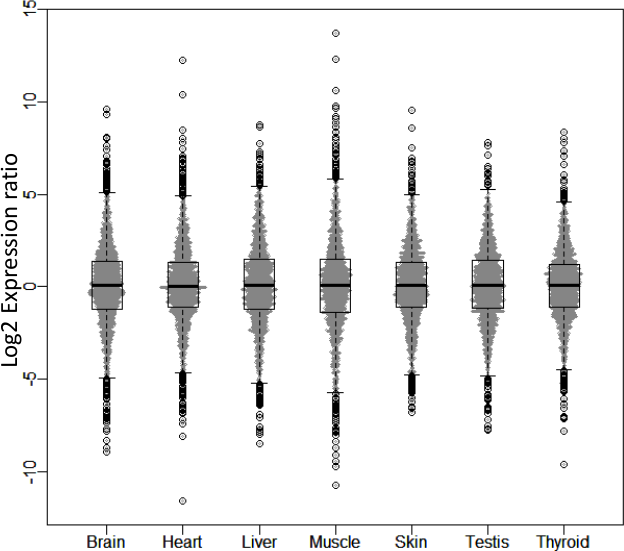

Supplement: S5 Fig — Shown are the expression ratios for 2,257 unique paralog pairs (not filtered for causal genes) in the seven disease-related tissues. The median ratios between paralogs observed per tissue ranged between 1.03–1.08 (namely ~0 on a log scale), suggesting that the general difference in expression patterns between paralogs is very small. (TIFF) [file pgen.1007327.s008.tiff]
